# Lack of association between the pancreatitis risk allele CEL-HYB and pancreatic cancer

**DOI:** 10.18632/oncotarget.15137

**Published:** 2017-02-07

**Authors:** Koji Shindo, Jun Yu, Masaya Suenaga, Shahriar Fesharakizadeh, Koji Tamura, Jose Alejandro Navarro Almario, Aaron Brant, Michael Borges, Abdulrehman Siddiqui, Lisa Datta, Christopher L. Wolfgang, Ralph H. Hruban, Alison Patricia Klein, Michael Goggins

**Affiliations:** ^1^ Department of Pathology, The Sol Goldman Pancreatic Cancer Research Center, The Johns Hopkins University School of Medicine, Baltimore, Maryland, USA; ^2^ Department of Surgery, The Sol Goldman Pancreatic Cancer Research Center, The Johns Hopkins University School of Medicine, Baltimore, Maryland, USA; ^3^ Department of Oncology, The Sol Goldman Pancreatic Cancer Research Center, The Johns Hopkins University School of Medicine, Baltimore, Maryland, USA; ^4^ Department of Medicine, The Sol Goldman Pancreatic Cancer Research Center, The Johns Hopkins University School of Medicine, Baltimore, Maryland, USA; ^5^ Department of Epidemiology, Bloomberg School of Public Health, Baltimore, Maryland, USA

**Keywords:** CEL, CELP, CEL-HYB, pancreatic cancer, chronic pancreatitis

## Abstract

CEL-HYB is a hybrid allele that arose from a crossover between the 3’ end of the Carboxyl ester lipase (*CEL*) gene and the nearby *CEL* pseudogene (*CELP*) and was recently identified as a risk factor for chronic pancreatitis. Since chronic pancreatitis is a risk factor for the development of pancreatic cancer, we compared the prevalence of the CEL-HYB allele in patients with pancreatic ductal adenocarcinoma to spousal controls and disease controls. The CEL-HYB allele was detected using Sanger and next generation sequencing. There was no significant difference in the prevalence of the CEL-HYB allele between cases with pancreatic ductal adenocarcinoma compared to controls; 2.6% (22/850) vs. 1.8% (18/976) (p=0.35). CEL-HYB carriers were not more likely to report a history of pancreatitis. Patients with pancreatic cancer are not more likely than controls to be carriers of the CEL-HYB allele.

## INTRODUCTION

Pancreatic ductal adenocarcinoma, more commonly known as pancreatic cancer, is the third-leading cause of cancer-related deaths in the United States with a 5-year overall survival of 8% [1]. This poor survival is related in part to the late presentation and rapid progression of disease [2]. A better understanding of the risk factors responsible for the development of pancreatic ductal adenocarcinoma, like chronic pancreatitis is needed not only to help identify individuals who might benefit from early detection strategies, but to refine our understanding of disease mechanisms. Patients with long-standing chronic pancreatitis have an increased risk of developing pancreatic cancer [3–6]. Among subjects with chronic pancreatitis, the risk of pancreatic cancer increases with increasing duration of pancreatitis and is highest among those with young-onset recurrent acute/chronic pancreatitis [7] as is seen in patients with inherited *PRSS1* mutations [8]. The risk of pancreatic cancer with chronic pancreatitis is higher among cigarette smokers [4] and may be greater among those who consume excess alcohol.

Although chronic pancreatitis is an important risk factor for pancreatic cancer development most of the mutated genes found to contribute to pancreatic cancer susceptibility when defective are classic tumor suppressor genes, such as *BRCA2, ATM, PALB2, p16*, and *STK11* [9]. Common low penetrance variants in gene loci including *ABO, TERT* and *PDX1* that contribute to pancreatic cancer risk have been identified through genome-wide association studies [10–14], but much of the familial clustering of pancreatic cancer remains unexplained. Recently, a large scale familial pancreatic cancer sequencing initiative reported the results of whole exome sequencing of 598 kindred and found preliminary evidence that mutations in the pancreatitis susceptibility gene *CPA1*, contributed to pancreatic cancer risk [15]. Deleterious mutations in *CPA1* were identified in four patients with familial forms of pancreatic cancer but not in controls [15]. Although *PRSS1* and *CPA1* variants have been linked to pancreatic cancer risk, to date variants in other chronic pancreatitis susceptibility genes such as *SPINK1* and *CFTR* have not been consistently found to be risk factors for pancreatic cancer [16–20].

Since much of the familial clustering of pancreatic cancer is not explained by germline alterations in known pancreatic cancer susceptibility genes [21–23], we evaluated whether gene variants associated with chronic pancreatitis could contribute to pancreatic cancer risk. Chronic pancreatitis encompasses a wide range of clinical and pathological findings [4, 24, 25] and while it is typically a clinical syndrome associated with chronic pancreatic inflammation and fibrosis, chronic pancreatitis can be clinically silent. Although it is the clinical syndrome of long-standing chronic pancreatitis that is the established risk factor for pancreatic cancer, it is possible that clinically silent chronic pancreatitis contributes to pancreatic cancer development. In this study, we determined if a recently described risk factor for chronic pancreatitis, “CEL-HYB”, might also be a risk factor for pancreatic cancer.

CEL-HYB is a hybrid allele (CEL-HYB) that has arisen from a crossing over event between the 3’ end of the carboxyl ester lipase (*CEL*) gene and the nearby *CEL* pseudogene (*CELP*) [26, 27]. *CEL* encodes carboxyl ester lipase which is expressed in the acinar cells of the pancreas [28] and in mammary glands [29]. Mutations in the variable number of tandem repeat (VNTR) region of *CEL* are a cause of young-onset diabetes (CEL-maturity onset diabetes of the young, CEL-MODY or MODY8) [30, 31, 32]. Molven et al. showed that CEL-HYB is significantly more prevalent in multiple different cohorts of European patients with chronic pancreatitis (2.6-4.3%) than in controls (blood donors) (0.7-0.9%) [26]. Here, we compared the prevalence of the CEL-HYB allele in a large series of patients with pancreatic cancer and in controls.

## RESULTS

### Detection of CEL-HYB alleles

**Figure 1:**
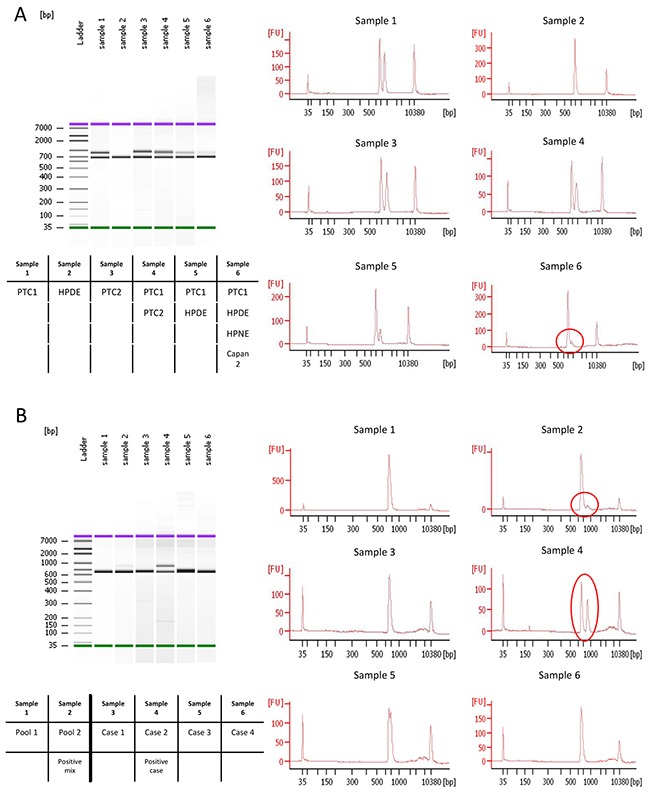
Bioanalyzer results of pooled 1^st^ PCR products **A.** HPDE showed only one band whereas the positive control, PTC1, had one extra band. Sample 6 (circle) shows the detection of an extra band when the positive control DNA was mixed with 3 negative control DNA samples. **B.** Examples of CEL-HYB positive and negative samples identified by Bioanalyzer. A pool mixed with one positive case and three negative cases showed two bands (Sample 2). Individual analysis of each of these four samples detected the positive case (Sample 4), and three negative cases (Sample 3, 5, and 6).

The Agilent 2100 Bioanalyzer system was used to identify CEL-HYB alleles. The CEL-HYB allele was readily detected by PCR amplification in the two positive control samples (PTC1 and PTC2) (as two bands representing wild-type (*CELP*) and CEL-HYB alleles) (Figure 1A), whereas PCR products from control samples (HPDE, HPNE, and Capan2 DNA) had only one band representing the wild-type (*CELP*) allele. An example of the CEL-HYB allele detected by Bioanalyzer analysis is shown in Figure 1B.

### Sequencing of CEL-HYB allele by NGS

**Figure 2:**
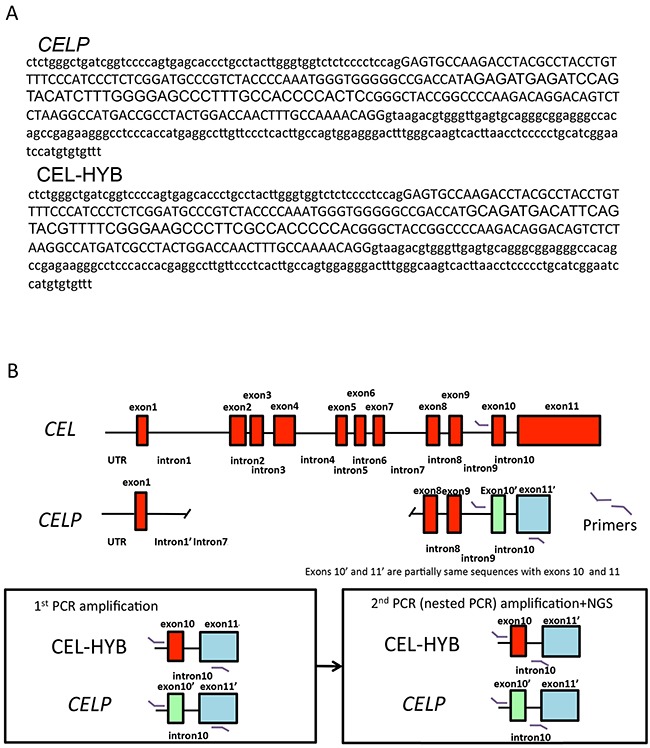
Detection of CEL-HYB by next-generation sequencing **A.** *CELP* and representative CEL-HYB sequence are shown respectively. The area highlighted in yellow the nucleotide differences between *CELP* and CEL-HYB. **B.** A schematic diagram of the *CEL* and *CELP* loci. The red box corresponds to each exon. The green box represents exon10’, and the blue box exon11’ of *CELP*. PCR primer sites for the PCR products generated for 1^st^ PCR and the nested PCR used in NGS are shown in the lower panel.

We used Sanger sequencing to localize the nucleotide sequence difference between the *CEL* P and CEL-HYB alleles (Supplementary Figure 1) and next-generation sequencing to sequence the CEL-HYB allele (Figure 2A and 2B). A schematic figure of the *CEL*, *CELP* and CEL-HYB allele and amplicons obtained by PCR amplification is shown in Figure 2B. Positive controls and samples with CEL-HYB amplicons identified by Bioanalyzer all had CEL-HYB sequences in ~50% of their reads by next-gen analysis whereas negative controls and samples without CEL-HYB amplicons had only the *CELP* sequence.

### CEL-HYB prevalence in cases and controls

We identified a CEL-HYB allele in 22 of 850 (2.6%) patients with pancreatic cancer, and 18 of 976 (1.8%) controls, including 7/400 (1.8%) of the healthy controls, 5/320 (1.6%) of the spousal controls and 6/256 (2.3%) other disease controls including 1 of 91 with a pancreatic neuroendocrine tumor (cases vs. controls, p=0.35) (Table 1). Only 1 CEL-HYB carrier reported a personal history of pancreatitis.

**Table 1:** CEL-HYB positive cases and disease controls

Case #	Diagnostic group	Primary diagnosis	Age	Sex	Race	Past medical history of pancreatitis
1	PDAC	PDAC	51	F	Caucasian	No
2	PDAC	PDAC	72	M	Caucasian	No
3	PDAC	PDAC	56	F	Caucasian	No
4	PDAC	PDAC	67	M	Caucasian	Yes
5	PDAC	PDAC	44	F	Caucasian	No
6	PDAC	PDAC	72	F	Caucasian	No
7	PDAC	PDAC	70	M	Caucasian	no
8	PDAC	PDAC	55	M	Caucasian	No
9	PDAC	PDAC	51	F	Caucasian	No
10	PDAC	PDAC	54	F	Caucasian	No
11	PDAC	PDAC	40	F	Caucasian	No
12	PDAC	PDAC	68	M	Caucasian	No
13	PDAC	PDAC	73	M	Caucasian	No
14	PDAC	PDAC	64	F	Caucasian	No
15	PDAC	PDAC	69	M	Caucasian	No
16	PDAC	PDAC	65	M	Caucasian	No
17	PDAC	PDAC	51	M	Caucasian	No
18	PDAC	PDAC	61	F	Caucasian	No
19	PDAC	PDAC	49	M	Caucasian	No
20	PDAC	PDAC	44	M	Caucasian	No
21	PDAC	PDAC	76	M	Caucasian	No
22	PDAC	PDAC	69	F	Caucasian	No
23	Disease controls	Duodenal adenoma	54	M	Caucasian	No
24	Disease controls	Lymphoepithelial cyst	57	F	Caucasian	No
25	Disease controls	Solid pseudopapillary neoplasm	31	F	Caucasian	No
26	Disease controls	Ampullary adenoma	79	F	Caucasian	No
27	Disease controls	Duodenal adenocarcinoma	62	F	Caucasian	No
28	Disease controls	Pancreatic neuroendocrine tumor	34	F	Caucasian	No
29-40	Healthy controls	Healthy controls				

## DISCUSSION

We found no statistically significant difference in the prevalence of CEL-HYB in patients with pancreatic ductal adenocarcinoma compared to controls. It should be noted that many pancreatic cancer risk variants are low-penetrant risk alleles [10–14] and we cannot rule out the possibility that CEL-HYB is a very low penetrant risk factor for pancreatic cancer development. Interestingly, Molven et al. found that the CEL-HYB allele has likely arisen through multiple independent recombination events since there was only limited linkage to nearby SNPs. This finding would indicate that the CEL-HYB allele would not be reliably reconstructed by imputing nearby SNPs and therefore may be easily detected using the SNP-based assays used for GWAS studies.

One notable finding in our study is that the prevalence of the CEL-HYB allele was higher in our control population (1.8%) than in the European population studied by Molven et al (~0.8%) [26]. This probably reflects differences in the prevalence of this allele in different populations and highlights the need to account for ethnicity when comparing cases and controls. The finding that only one of the CEL-HYB carriers with pancreatic cancer reported a clinical history of chronic pancreatitis highlights the low-penetrant nature of CEL-HYB. Since the clinical syndrome of chronic pancreatitis is uncommon, it is likely that most CEL-HYB carriers do not develop pancreatitis. Interestingly, a recent study found that no association between a different CEL-HYB allele found in Asian populations and CP. [33] However, unlike the European version, the Asian CEL-HYB variant is suspected to be functionally inactive. Further research is needed to determine the risk factors responsible for the development of chronic pancreatitis in CEL-HYB carriers.

In conclusion, we find no evidence for an association between the CEL-HYB allele and likelihood of developing pancreatic cancer.

## MATERIALS AND METHODS

### Patients and specimens

This study included patients who underwent pancreatic resection at the Johns Hopkins Hospital, spousal control individuals enrolled in the National Familial Pancreas Tumor Registry (NFPTR) at Johns Hopkins, and individuals enrolled in an inflammatory bowel disease study. The individuals included i) 850 patients (mean age 65 ± 10.6 years, 453 males, 753 Caucasian, 52 African-American) who underwent pancreatic resection for clinically sporadic pancreatic ductal adenocarcinoma, ii) 320 spouses (without pancreatic cancer or pancreatitis) of individuals enrolled in The National Familial Pancreas Tumor Registry (NFPTR), iii) 400 healthy relatives participating in a study of inflammatory bowel disease, v) 256 individuals who underwent pancreatic resections for diseases unrelated to pancreatic ductal adenocarcinoma including patients who underwent pancreatic resection for a pancreatic neuroendocrine tumor (n=91), duodenal cancer (n=51), duodenal adenoma [12], ampullary adenoma [13], gall bladder cancer [7], carcinoid or GIST [17] other non-periampullary cancer [23], serous cystadenoma [25], solid pseudopapillary neoplasm [5], other non-neoplastic non pancreatic diseases [12]. DNA from spousal controls was isolated from peripheral blood mononuclear cells. DNA from cases and controls who underwent pancreatic resection was isolated from stored fresh-frozen normal tissue (duodenum, spleen, or pancreas) as previously described [10]. The clinicopathological characteristics of the cases and disease controls including their final diagnosis are presented in Table 1. All elements of this study were approved by the Johns Hopkins Institutional Review Board and written informed consent was provided from all patients.

### DNA extraction

Genomic DNA was extracted using the QIAamp DNA Micro Kit (Qiagen) according to the manufacturer's instructions. DNA samples were quantified using the Quantifiler Human DNA Quantification kit (Applied Biosystems).

### CEL-HYB assay

The *CEL*/*CELP* locus was amplified using Integrated DNA Technology (IDT) PCR primers; sense: 5’ – gtctctgggctgatcggtc – 3’ and antisense: 5’ – cagactcggagttgcctgtc – 3’. SYBR green-based polymerase chain reaction (PCR) assays were used to differentiate wild-type alleles from CEL-HYB alleles. Two positive control samples (PTC1 and PTC2) were generously provided by Dr. Molven [26] and negative control samples (DNA from cell lines HPDE, HPNE, and Capan2) were used to validate the assay. The wild-type primers amplified the *CEL* pseudogene (*CELP*) when no CEL-HYB was present and amplified both alleles when CEL-HYB was present. The reaction mixtures (20 µl) consisted of 10 µl SYBR Green Master Mix (Qiagen), 0.2 µl of each forward and reverse primers (primer set1: final concentration 0.1 μM), and 2 µl genomic DNA of 6 ng/ul with nuclease-free water. Amplification was performed using the SimpliAmp™ Thermal Cycler (ThermoFisher scientific) at the following cycling conditions: started with a denaturation step at 95°C for 12min which was followed by 56 cycles of 95°C for 20sec, 60°C for 40sec, and 72°C for 90sec and with a final extension at 72°C for 2min. PCR products were subsequently electrophoresed using an Agilent 2100 Bioanalyzer (Agilent Technologies, Loveland, CO USA) using the Agilent High Sensitivity DNA Kit following the manufacturer's protocol. We found that CEL-HYB alleles could be detected mixtures of PCR products from 4 individuals were pooled. Pooled samples containing CEL-HYB alleles were then run individually to identify the sample containing the allele. CEL-HYB alleles were confirmed using next-generation sequencing (NGS). Sanger sequencing (performed at the Johns Hopkins DNA sequencing Core) was used to identify the common and unique sequences between CEL-HYB and CELP.

### Next-generation sequencing (NGS)

The libraries for NGS were prepared from a nested PCR; sense: 5’- adaptor sequence – gtctctgggctgatcggtc – 3’ and antisense: 5’ – aaacacacatggattccgatg – 3’, which started with a denaturation step at 94°C for 3min, followed by 40 cycles of 94°C for 30 sec, 58°C for 30sec, and 68°C for 1min. Libraries were cleaned up using Agencourt AMPure XP Reagent (Beckman Coulter) on a magnet stand, eluted into low TE buffer and subsequently quantified using the Ion Quantification Kit (Life Technologies) following the manufacturer's protocols. The libraries were introduced with emulsion PCR reagents into the Ion OneTouch2 system (Life Technologies) for about 5 hours, where the libraries were ligated to Ion Sphere Particle (ISPs). The ISPs with the libraries were cleaned up and enriched in the Ion OneTouch ES (enrichment system) (Life Technologies) and subsequently loaded into an Ion Torrent Personal Genome Machine (PGM, Life Technologies) for sequencing. Post-sequencing data analysis, including alignment to the hg19 human reference genome, was done using the software Geneious (version R9, Auckland, New Zealand).

### Statistics

The Chi-square test was used to compare the prevalence of CEL-HYB variants in cases vs. controls. SPSS software was used (v22, IBM). A 2-tailed *p* < 0.05 was considered statistically significant.

## SUPPLEMENTARY MATERIALS FIGURE



## Abbreviations

CEL: carboxyl ester lipase
CELP: carboxyl ester lipase pseudogene
CEL-HYB: CEL hybrid allele
PDAC: pancreatic ductal adenocarcinoma
PCR: polymerase chain reaction
PTC: positive control, NGS: next generation sequencing
VNTR: variable number tandem repeat
SNP: single-nucleotide polymorphism.
