# Supplementary material for: Lack of association between the pancreatitis risk allele CEL-HYB and pancreatic cancer
Source: Oncotarget. 2017 Feb 7;8(31):50824–31. doi: 10.18632/oncotarget.15137 (PMC5584208; doi:10.18632/oncotarget.15137)
Supplement: Supplementary file 1 [file oncotarget-08-50824-s001.pdf]

Lack of association between the pancreatitis risk allele CEL-HYB and pancreatic cancer

SUPPLEMENTARY FIGURE

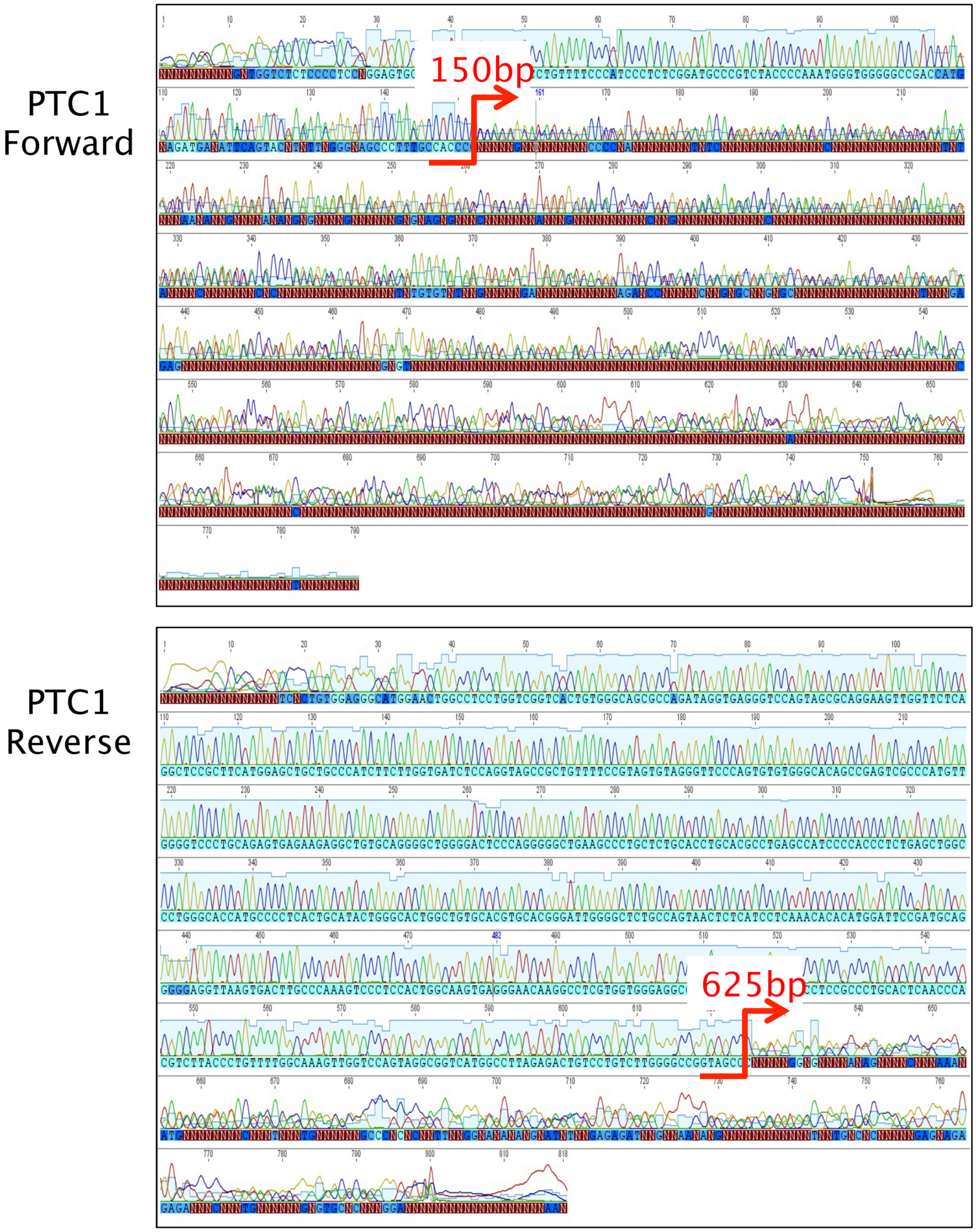

Supplementary Figure 1: Sanger sequencing results of 1st PCR product of PTC1. The CEL-HYB sequence is located in the unreadable sequence starting at 150bp in the forward trace, and 625bp in the reverse trace.
